# Factors that influence the preferences for telehealth among family caregivers of disabled elders: a qualitative study

**DOI:** 10.1016/j.ijnsa.2025.100351

**Published:** 2025-05-21

**Authors:** Lina Guan, Guijuan Zhang, Yuyang Yi, Yaqin Liang, Xiaochun Zou, Bingsheng Guan, Yanya Chen, Wai-kit Ming

**Affiliations:** a School of Nursing, Jinan University, Guangzhou, PR China; b Department of Infectious Diseases and Public Health, Jockey Club College of Veterinary Medicine and Life Sciences, City University of Hong Kong, PR China; c Shaoguan University, PR China; d School of Health, Dongguan Polytechnic, Dongguan, PR China; e The First Affiliated Hospital of Jinan University, Guangzhou, PR China; f Institute of Global Governance and Innovation for a Shared Future, City University of Hong Kong, PR China

**Keywords:** Aged, Frail elderly, Caregivers, Qualitative research, Telehealth

## Abstract

**Background:**

Family caregivers of the disabled elderly lack adequate support in their caregiving process. Telehealth has significantly alleviated the burden on family caregivers, both subjectively and objectively. It is currently utilized in family care settings for various populations, including disabled elderly individuals and children. Nevertheless, the use of telehealth among family caregivers for disabled elderly people is limited, and the factors that affect this phenomenon are not yet fully understood.

**Objective:**

The goal of this qualitative research was to explore the factors affecting family caregivers' preferences regarding telehealth services for disabled elderly individuals.

**Methods:**

This qualitative research employed semi-structured interviews. Four focus group interviews were conducted with 20 family caregivers of disabled older people. Interviews were recorded and transcribed verbatim. The results were analyzed through thematic content analysis.

**Results:**

The study found that there were two main factors influencing preferences: barriers of carergivers' use of telehealth, and facilitators of carergivers' use of telehealth. Barriers of carergivers' use of telehealth included learning capacity, acceptance of telehealth, differences in treatment modalities, health self-management capability, habituation to medical care, job, cost, limitations, operation experience, quality of care in nursing homes, convenience of medical treatment and popularisation. Facilitators of carergivers' use of telehealth included convenience and professionalism and authority of healthcare professionals.

**Conclusions:**

This study identified a series of factors that appear to influence the preference of family caregivers of elderly individuals with disabilities for telehealth in the context of conducting home care. These factors should be considered when designing and developing telehealth for conducting home care.

What this paper adds from an international perspective?

• This study provides deeper insights into the factors influencing telehealth preferences among family caregivers of disabled elderly, particularly in non-Western contexts, addressing a gap in existing international research.
• By identifying barriers and facilitators in the use of telehealth from the family caregivers' standpoint, this study offers international researchers and policymakers new avenues for developing targeted telehealth interventions.

How this study is increasing our understanding?

• This study explores the barriers and facilitators of telehealth use from the caregiver's perspective, enhancing understanding of their specific needs in caregiving.
• The findings help inform the design of more accessible, user-friendly telehealth platforms and policies tailored to the needs of family caregivers of disabled elderly individuals.

## 1 Introduction

According to a report by the World Health Organization (WHO) on disability, it is estimated that 10.2 % of the population aged 60 and over across 194 countries and territories globally experience some form of disability (Seelman, 2011). The disability rate of the elderly in China is between 10.48 % and 13.31 % (Zhang and Wei, 2015). Aging in place is still the main way to age, with the majority of older people with disabilities preferring to stay at home and 95 % receiving some level of care from family and friends (Benefield and Beck, 2007). Family caregivers were typically close relatives, such as spouses and adult children, who lacked specialized training (Christian and Wilson et al., 2023). Family caregivers are often involved in supporting older people throughout their care following hospitalization (Nuckols and Keeler et al., 2017). They have significantly contributed to enhancing the quality of life, supporting functional independence, delaying functional decline and institutionalization, and saving healthcare expenditures among older adults (Del-Pino-Casado and Priego-Cubero et al., 2021). Nevertheless, caregivers often put the requirements of those they look after before their own personal wishes and lifestyle preferences (Riffin and Van Ness et al., 2019). Hence, half of family caregivers reported experiencing some form of care overload (Cheng, 2017), while 17 % experienced severe health problems, especially neuropsychiatric disorders (Chi and Demiris, 2015), which further impairs their caregiving capacity (Chary and Hernandez et al., 2024). The lack of effective models of care for family caregivers also contributes to the high burden and low quality of care they receive (Yue and Jia et al., 2022). Therefore, there is an urgent necessity for the implementation of home care interventions aimed at enhancing the standard of living among family caregivers of disabled elders, as well as enhancing the quality of care provided by them.

Telehealth involves delivering healthcare services remotely using telecommunications like phone calls, video conferencing, texting, and internet platforms (Yang and Li et al., 2024). Telehealth enhances health education, aids in decision-making processes, fosters the cultivation of problem-solving abilities, while also providing invaluable social support. Additionally, it enhances efficiency and saves resources and time for informal caregivers, family caregivers included. Telehealth played a significant role in alleviating the burden on family caregivers, both subjectively and objectively (Miyatake and Kosaka et al., 2021). Currently, it has been applied in family care, including the disabled elderly and children (Guo and Xu et al., 2024). Family caregivers may benefit from professional guidance and support delivered via telehealth, resulting in enhanced self-efficacy and improved quality of care (Slaboda and Nelson et al., 2021). However, the utilization of telehealth by family caregivers of elderly individuals with disabilities is limited (Hautala and Keränen et al., 2017). At the same time, older people with disabilities and their family caregivers in China face serious challenges when using telehealth (Zihao and Raija et al., 2019). When using telehealth, the perceived usefulness is assessed to be high in family caregiving of elderly and disabled subjects (Hautala and Keränen et al., 2017). However, to the best of our knowledge, no studies have explored the factors that influence whether family caregivers of disabled elderly individuals use telehealth.

## 2 Research aim

The aim of this qualitative research was to explore the factors affecting family caregivers' preferences regarding telehealth services for disabled elderly individuals. Specifically, this study aimed to address the following research questions: (1) What are the barriers that hinder family caregivers from using telehealth in providing care for disabled elderly individuals? (2) What are the facilitators that encourage family caregivers to adopt telehealth services in their caregiving process?

## 3 Methods

This qualitative study was conducted within an interpretivist paradigm, which posits that reality is socially constructed and subjective. This epistemological stance guided our decision to use focus group interviews to explore the nuanced perspectives of family caregivers regarding telehealth. The data consisted of semi-structured interviews with family caregivers of older people with disabilities and were analyzed thematically. The group of authors comprises an interdisciplinary team of researchers, all specializing in qualitative research. COREQ guidelines were followed in describing the study design, analyzing, and presenting the results of the study (Tong and Sainsbury et al., 2007).

### 3.1 Participants and study design

Participants were recruited through purposive sampling, where individuals with appropriate experiences or characteristics were consciously selected (Patton, 2015), and ultimately family caregivers of the disabled elderly in a community in Dongguan City were selected for focus group interviews. No individual in the focus group team was previously related to the participant. A poster was released via WeChat with a brief description of the study and instructions to contact the researcher via the contact information in the poster if they wished to participate in the study. All participants will sign an informed consent form.

Qualitative research provides deep insights into participants' experiences and perspectives, allowing for a rich understanding of complex issues in their real-life context. Compared to one-on-one interviews, focus group interviews are based on the premise that group interaction enhances the expression and clarity of participants' views. This is because individuals often feel safer and less anxious when discussing difficult experiences in a group setting. Therefore, this study used focus group interviews to investigate the factors influencing the use of telehealth by family caregivers of elderly people with disabilities, where interaction, dialogue and shared reflection between participants within the group could increase and deepen understanding of the phenomenon being explored.

Inclusion criteria were as follows: (1) family caregivers should be at least≥18 years of age; be a family member of a disabled older person (age≥60 years, Katz Index score < 6) who has cared for the older person as a primary carer for >3 months; (2) without self-reported diagnosed physical or mental disease; (3) voluntary participation. Prospective participants received an invitation letter elucidating the purpose of the study and were subsequently queried regarding their willingness to participate in the research. Eventually, 20 participants (i.e., 11 males and 9 females) fulfilled the study's inclusion requirements and took part in the research. Our research team believes that data saturation has been reached in this pioneering environment. After the interview, participants received a small box of tissues as a thank-you gift. According to previous literature (Braun and Clarke, 2006), the number of participants in focus group interviews generally ranges from five to six individuals.

### 3.2 Data collection

Data was collected in July and August 2024. Individuals interested in participating in the study were instructed to contact the primary researcher to schedule data collection, which was to be conducted in a silent and private conference room. The main researcher, assistant, and field note-taker arrived at the meeting room 30 min before the participants arrived to arrange seating and prepare recording equipment. The main researcher involved in the evaluation holds a doctoral candidate in public health and has over 4 years of experience in conducting similar studies. The assistant has a Master's degree and has participated in several research projects related to this topic. The field note-taker is an experienced researcher with a Bachelor's degree and has been trained specifically for this study to ensure accurate and reliable data collection. The participants had the option to sit comfortably in a circle, where information could be conveyed via facial expressions and body language. Participants were explained the objectives of the study, the method of data collection and voluntary participation in the study. Focus group interviews were conducted in Mandarin by four researchers using a semi-structured interview format, and all sessions were recorded (guiding questions are listed in Table 1). A semi-structured focus group interview guide was developed based on the literature review, and the purpose of the study. The questions included in the discussion guide were similar to those used in the individual interviews, but were further adapted from the experience of the individual interviews to suit the different conditions of the participants. The interview was divided into two separate parts. In the initial section, participants explained their perceptions of previous approaches they used to solve caring problems. In the next section, the researcher gave a concise overview of the present situation of telehealth within the care sector for disabled elderly individuals, ensuring that all participants gained a basic and comprehensive understanding. Subsequently, the interviewees were afforded the opportunity to express their attitudes, expectations, and ideas regarding the implementation of telehealth within home care. As the data had reached saturation, we decided not to recruit other participants after interviewing 20 participants. The interviews, each lasting for 40 min approximately, were recorded on audio and then transcribed by a professional transcription service and one researcher.

**Table 1:** Focus group outline.

Number	Question
1	Please share your feelings about previous family caregiving methods
2	Have you experienced something related to telehealth?
3	If telehealth could be used to address care-related issues, would you like to try it?
4	If telehealth could be used to address care-related issues, what factors would you consider important?
5	Do you have any suggestions on when telehealth should be used to address care-related issues?
6	Is there any supplementary information you wish to incorporate into the interview content?

### 3.3 Data analysis

Inspired by Braun and Clarke's six-step analysis (Dilorio and Hockenberry-Eaton et al., 1994), an inductive thematic approach was used to analyse the data. A thematic approach means that researchers focus on interpretation and creativity based on an understanding of data as “context-bound, positioned, and situated” (Braun and Clarke, 2019). 1) Familiarise yourself with the data. 2) Create the source code. 3) Develop preliminary themes. 4) Review the themes. 5) Define and name the themes. 6) Write the report. We used NVivo 14 software to organize the data. To improve the validity of the analyses, member checking was used. Following initial coding and theme development, participants were invited to review the findings to ensure that their views were accurately expressed. Their feedback helped refine the themes and ensure the credibility of the interpretations. One of our researchers carefully reviewed the transcripts to ensure that the nuances of the participants' responses were accurately captured. Two researchers coded both transcripts to develop an initial coding framework. After discussing and revising the framework, one author coded all of the remaining texts and created categories. When the initial codebook has been sufficiently calibrated through multi-coder collaboration, a single coder can more efficiently maintain logical consistency, avoiding redundancy caused by later disagreements between multiple coders (Boyatzis, 1998). To ensure that all transcripts were coded consistently, these researchers met regularly to further discuss the results of the analyses, create codes, and categorise transcripts. Subsequently, themes emerged based on patterns of meaning in the data and consensus was reached among the primary research team.

We followed the confidence, transferability and conformity criteria used by Lincoln and Goba (Lincoln and Guba, 1985). Credibility is sustained through a systematic approach, transparency of methodology, iterative discussions among researchers, exploration of different perspectives, and a reflective attitude. A reflective strategy was used throughout to maintain and enhance methodological rigour, which is necessary to achieve reliable results. Data analyses included thorough comparisons and discussion until a consensus was reached. The research team provided rich descriptive data as well as excerpts from the interview guide to ensure transferability.

### 3.4 Ethical considerations

Ethical approval was obtained from the Institutional Review Board of Jinan University (Reference number: JNUKY-2023–0128). Participation in the study was strictly voluntary. Interviews were solely conducted after obtaining the informed consent of all participants. The research objectives, methodologies, and procedures were thoroughly explained to the participants prior to their involvement. Participants were reassured of their right to withdraw from the study at any time, without facing any obstacles. Participants will receive tissue paper worth RMB 50 as a token of appreciation. In order to protect the privacy and confidentiality of the participants, the datasets generated and/or analysed in this study are not publicly available but can be obtained from the corresponding author.

## 4 Results

A total of 20 participants (comprising 11 males and 9 females) with a mean age of 50 years (SD = 11.94) were enrolled in the study (Table 2). Of these participants, 20 % were sons of disabled older adults, 10 % were daughters, 65 % were spouses, and 5 % were categorized as others. The research employed focus group interviews, which were conducted in four distinct groups of participants. Through this process, two overarching themes were identified: (1) barriers of carergivers' use of telehealth, (2) Facilitators of carergivers' use of telehealth. These main themes were further disaggregated into 14 subthemes for a more comprehensive analysis (Table 3).

**Table 2:** Demographics table.

Participant code	Age	Gender	Marital status	Work status	Relationships with older adults
1	32	male	married	in business	son
2	42	male	married	surgeon	son
3	43	male	married	in business	son
4	45	male	married	teacher	son
5	46	male	married	retirement	spouse
6	51	male	married	unemployment	spouse
7	52	male	married	unemployment	spouse
8	53	male	married	unemployment	spouse
9	57	male	married	unemployment	spouse
10	59	male	married	unemployment	spouse
11	25	male	unmarried	unemployment	niece
12	48	female	married	teacher	daughter
13	52	female	married	in business	daughter
14	60	female	married	retirement	spouse
15	54	female	married	retirement	spouse
16	58	female	married	unemployment	spouse
17	62	female	married	unemployment	spouse
18	63	female	married	unemployment	spouse
19	65	female	married	unemployment	spouse
20	66	female	married	unemployment	spouse

**Table 3:** Main themes and subthemes from the focus group interviews.

Main themes	Subthemes
Barriers of caregivers' use of telehealth	Learning capacity
	Acceptance of telemedicine
	Differences in treatment modalities
	Health self-management capability
	Habituation to medical care
	Job
	Cost
	Limitations of telehealth
	Operation experience
	Quality of care in nursing homes
	Convenience of medical treatment
	Popularisation
Facilitators of caregivers' use of telehealth	Convenience.
	Professionalism and authority of healthcare professionals

### 4.1 Barriers of carergivers' use of telehealth

#### 4.1.1 Learning capacity

Four family caregivers expressed their low self-perceived learning ability regarding smart devices such as mobile phones, resulting in their inability to complete online telehealth operations even with clear operational prompts. Additionally, they expressed concern that their need to learn new skills might become a burden on other family members, leading them to autonomously choose to avoid learning and trying out new things like telehealth. In practical applications of telehealth, the deficiency of learning ability about these new e-skills among family caregivers prevents them from enjoying the convenience it offers. Even if they attempt to use telehealth, their low learning ability hinders smooth communication with medical staff, leading to feelings of frustration and helplessness during the process.

"I don't have that level of skill, it's not that I don't want to try it, it's that I can't figure it out. Every time, there are so many rules and regulations that pop up, I don't understand what it (the web) is talking about; it makes me feel very confused. I can’t use telehealth as smoothly as younger people do. My kids live far away and have to work, I don't want to trouble them."

"My own education level is not high enough. I tried Baidu medical consultation, but I don't understand the questions he asked and can't answer them (doctors online), it would be more beneficial to proceed with a visit to the hospital. "

#### 4.1.2 Acceptance of telehealth

Respondents were often unclear about the qualifications, purpose, usefulness, and benefits of telehealth resources, resulting in low acceptance of telehealth among participants. Due to the compulsory requirement for identity verification when using telehealth, some respondents were concerned about privacy breaches, doubting both the authenticity and effectiveness of telehealth and fearing they might be scammed. Advertisements even pop up during the use of telehealth, which some respondents who have tried telehealth found off-putting. Therefore, they only consider telehealth as an auxiliary reference for diagnosis and treatment.

" It has always been my contention that telehealth is merely an auxiliary means. In terms of practical application (of home care), it is evident that direct, face-to-face interaction remains the optimal choice. The abundance of advertisements popping up when you open it (the website or application), which are very off-putting and untrustworthy. Sometimes I don't even know who the other person is, if they're a scammer. They chat with me online, and I don't know what kind of person they are. Moreover, Then I’m asked to enter my Identity Document number and verification code, which makes it feel unreliable. "

#### 4.1.3 Differences in treatment modalities

The perspectives and methodologies of family caregivers with regard to treatment varied considerably. Two of the interviewees viewed medication as the sole form of treatment, thereby neglecting the importance of comprehensive therapy and self-management. Consequently, they no longer pursued additional avenues, including telehealth, to address issues arising from their care receivers’ illnesses. Instead, they were content with maintaining the current status as the optimal outcome for treatment and recovery. Conversely, two other interviewees preferred to rely on nursing homes and were prepared to delegate the majority of responsibilities pertaining to the treatment of the illness and the care of the elderly to the medical staff in such facilities.

" For conditions like diabetes and hypertension, it's common for elderly people to take medication regularly as part of their treatment. As for some other aspects, I might not be able to handle them effectively since I'm not an expert in that field. Even if telehealth is used, it probably won't be very helpful; it would be better to send them to a nursing home where there are professionals."

#### 4.1.4 Health self-management capability

Health self-management capability describes the ability of family caregivers and their care receivers to independently manage minor health issues. It encompasses the use of personal experience, self-care practices (such as purchasing over-the-counter medications), and leveraging online resources for health information. Some of the interviewees indicated that both they and their elderly family members maintain good physical health and rarely suffer from illnesses in their daily lives. Even when confronted with minor health issues like the common cold, they demonstrated strong self-management skills. In such cases, they tend to rely on their life experiences to inform their actions. For instance, they may autonomously purchase over-the-counter medications based on past experiences to alleviate symptoms. Additionally, they leverage modern technology, such as searching the internet for relevant information, to access more professional health guidance or solutions.

" In many cases, minor health issues of the elderly can be effectively addressed through self-care, including the acquisition of necessary medications. Nowadays, the internet provides great convenience, allowing us to quickly search for information and find out how to handle certain problems. "

#### 4.1.5 Habituation to medical care

Habituation to medical care refers to the established, routine reliance on traditional, in-person healthcare. Some family caregivers maintained trust and habit in the traditional model of seeking medical care, which involves face-to-face communication with healthcare professionals to obtain health guidance. Even knowing the advantages of telehealth, they would still prefer to make appointments in person. In their view, this direct interaction made them feel more substantial and was something that telehealth could not replace. Additionally, the utilization of medical insurance cards in a non-digital capacity had been a long-established practice among this demographic. A considerable number of interviewees expressed uncertainty regarding the extent to which their medical insurance coverage encompasses telehealth services.

" Currently, we do not have the habit of using the internet for healthcare purposes and have not considered this avenue. We are still more accustomed to the traditional face-to-face approach of taking our elderly family members to the hospital. Although it may be more tiring and involve waiting in lines, we find it comforting and feel a sense of intimacy in the communication, which is something that cannot be achieved online."

"I can use my medical insurance card to buy medicine at the hospital directly, but if I consult a doctor online for my father, I can't use my card."

#### 4.1.6 Job

The two family caregivers who worked in the nursing home benefited from the distinctive work environment, which facilitated close contact with the attending physicians and nurses. This facilitates convenient access to essential medical data, including case records, test results, and medication guidance. Consequently, the utilization of telehealth is not a priority for them.

"If I have any questions, I ask them (colleagues, i.e., doctors and nurses) directly, because there are many acquaintances in the hospital, it's convenient to ask, and it saves time compared to researching using telehealth on my own. I'm aware of these medical apps, but I haven't used them yet and don't feel that I need to use them."

#### 4.1.7 Cost

Half of participants felt that the cost of telehealth was too high and beyond their financial means. In certain instances, the cost incurred was found to exceed expectations, particularly in comparison to traditional offline registration and consultation fees. In the event that telehealth did not provide a satisfactory resolution, patients might be compelled to re-register offline for a subsequent consultation, effectively doubling the financial burden associated with medical care. This perceived burden, in turn, has had a notable impact on their willingness to utilize telehealth services.

"I've tried it before, like using the online consultation service at the Third Affiliated Hospital of Sun Yat-sen University. Many people found the fees unreasonable. Just a few questions would cost around 50 yuan, and if you ended up needing to go to the hospital afterward, it became a double cost. It's not as convenient or cost-effective as just taking someone to the hospital directly. "

"I had a bad experience with online consultation. I tried it once, but I was charged just for opening the app, so I stopped using it. I prefer to choose a more economical way to access healthcare services."

#### 4.1.8 Limitations of telehealth

Caregivers perceived that telehealth evaluations were not exhaustive or individualized, leading to a certain degree of one-sidedness in diagnosis results. In such a scenario, the formulation of treatment plans relied more heavily on the doctor's experience rather than on a comprehensive and individualized assessment. This also became one influencing factor affecting family caregivers of disabled elders in using telehealth.

"Telehealth definitely cannot achieve the same level of effectiveness as traditional in-person consultations, where doctors follow the ancient principles of 'observation, auscultation and olfaction, interrogation, and palpation.' Without physically examining the patient, the effectiveness is greatly reduced. Moreover, in traditional settings, we would revisit the doctor for medication adjustments based on the patient's progress. Remote consultations, however, often rely solely on experience and may continue prescribing the same medication without considering potential changes in the patient's condition."

#### 4.1.9 Operation experience

Approximately half of the respondents believed that telehealth apps or websites still had areas that needed improvement. Firstly, respondents expressed a preference for a simple and intuitive interface that would facilitate rapid access to the consultation process. Secondly, respondents indicated a preference for a telehealth operating interface that supports a variety of language services, including but not limited to multiple ethnic languages and local dialects. Thirdly, respondents expressed the desire for a dedicated professional team to oversee the operation of the telehealth system. The objective of this team would be to optimize service processes, reduce patient waiting times and, most importantly, ensure that the privacy of patients and their caregivers is maintained.

"Be concise, to avoid unnecessary operations, both voice and video are acceptable, don't beat around the bush. Sometimes you also need a verification code and Identity Document number, my first reaction is whether he is trying to scam me for money… If there was a doctor on duty like in a clinic, it would be more convenient… In current telehealth, when I have an online consultation, the doctor on the other side may not have the time to respond to me immediately. Additionally, supporting Cantonese would be essential since my Mandarin is limited, and misunderstandings can occur (when I use telehealth)."

#### 4.1.10 Quality of care in nursing homes

Respondents indicated that they noticed that modern nursing homes not only have professional and experienced medical staff, but also have a clean and comfortable care environment. They benefit from the holistic care model provided by these homes, which greatly reduces the burden on family caregivers. Some of the respondents clearly expressed their wish to send the disabled elderly to nursing homes for care. In their view, even with the help of new technologies such as tele-health, their own non-professional caregiving capacity can hardly match the professional knowledge and skills of medical professionals in nursing homes. In their view, telehealth cannot address the core care challenges faced by family caregivers at the root. The reason why the quality of nursing home care affects carergivers' preference for telehealth is that when the quality of nursing home care is high, family caregivers will rely more on professional care in nursing homes and have less need for telehealth-assisted home care; conversely, if the quality of nursing home care is poor, family caregivers may look to telehealth to make up for their own caregiving deficits, and thus have more of a preference for telehealth.

"My friend's parents have also been to a nursing home, and they like it (nursing home); there are many activities in the nursing home, and they are very happy…Perhaps sending the elderly to a nursing home is more practical than using any telehealth."

#### 4.1.11 Convenience of medical treatment

The majority of respondents stated that the current comprehensive pension policy has resulted in the provision of an array of medical equipment and resources at community service centers and health stations. And, the proximity of these facilities to local hospitals and residences, coupled with the ease of transportation, has significantly reduced the time and cost associated with medical treatment for patients. Additionally, local medical institutions frequently provided complimentary medical services, including free clinics and physiotherapy, which enhanced the accessibility of medical care. This also influenced their utilization of telehealth to a certain degree.

"We have doctors in our town, so we just go to see the doctor directly. There's a health station in the village, so we can solve small problems there, and we don't consider anything like online medical services. We only go to the hospital when we have big problems."

"The hospital is very close to my home, I can go to the hospital in 15 min."

"Some family members have gone for physical therapy (at the town health service station), once a week, and it's free of charge."

#### 4.1.12 Popularisation

Almost all respondents expressed a notable lack of familiarity with telehealth, a contemporary medical model. The respondents demonstrated a lack of awareness of the potential of telehealth to address practical health concerns and exhibited limited understanding and access to this modality. Even when they occasionally heard about telehealth, they might be unable to seamlessly transition into using it due to limited access channels and unclear operational guidelines. Some respondents expressed the hope that relevant institutions would intensify their promotional efforts to disseminate knowledge and advantages of telehealth to every household.

"I really don't know if there is any online way to consult a doctor. I only know that I can register online, and no one has told us about this."

### 4.2 Facilitators of carergivers' use of telehealth

#### 4.2.1 Convenience

Telehealth has been demonstrated to have beneficial effects and can be employed as an efficacious management instrument for non-emergency and non-critical illnesses. It offered timely medical advice to the elderly during critical moments. For family caregivers who were geographically dispersed or had mobility issues, telehealth provided a way to communicate, enabling them to remain apprised of the health status of their loved ones at all times. For elderly individuals with mobility issues or residing in remote areas, telehealth reduces the time and discomfort associated with travel, thereby enhancing the accessibility of healthcare services.

"My workplace is over 40 km away from home, so if there's an emergency at home, I can't get back quickly. Having a medical app is very convenient for me. I can just make a call and know the situation of my family members. If it's not urgent, like just skin irritation, I can also consult a doctor online without having to make a special trip to the hospital."

" The convenience (of telehealth) is undeniable. In the past, whenever there was an issue, I had to go to the hospital specifically, register, and then find a doctor. Now, with technology, it's much more convenient, cost-effective, and time-saving.

#### 4.2.2 Professionalism and authority of healthcare professionals

Some respondents identified the professionalism and authority of healthcare providers as important factors influencing their trust in telehealth. It was stated that the credibility of telehealth platforms was enhanced when they were endorsed by authoritative institutions or reputable hospital teams. This, in turn, would increase the willingness of patients to utilize telehealth services.

"Firstly, the qualifications of the internet hospital must be impeccable, and the hospital platform should be well-established and reputable. Only then will I trust and be willing to use it. However, if it's just a small clinic or an unknown hospital, I would definitely have doubts about its credibility."

## 5 Discussion

Telehealth offers benefits such as enhanced access and better healthcare quality for patients interacting with providers, alongside reductions in expenses and time spent (Triantafillou and Layfield et al., 2021). Throughout the COVID-19 outbreak, the use of telehealth was rapidly expanding across the healthcare spectrum (Mann and Chen et al., 2020). For instance, the utilization of telehealth in pediatric healthcare was increasing, encompassing prevention, disease management, remote monitoring, home-based care, diagnostic and therapeutic support, as well as personalized assistance programs (Zuccotti and Bertoli et al., 2020). Through telehealth, family caregivers of elderly individuals with disabilities could attain professional guidance and support, without geographical boundaries, scheduling clashes, or mobility constraints (Orlando and Beard et al., 2019). However, results have shown that telehealth use is low among the population of family caregivers of elderly people with disabilities. This qualitative study identifies, describes, and explains the factors that influence family caregivers of older adults with disabilities' preference for using telehealth for home care.

Learning ability was an important factor limiting the choice of family caregivers of elderly people with disabilities in using telehealth. The results of this study indicated that the majority of family caregivers for elderly individuals with disabilities were often older spouses who were reluctant to utilize telehealth for home care. Their perceived low learning capacity has contributed to a deficiency in digital literacy. Furthermore, older adults preferred passive learning to actively seeking guidance in accessing online health information (Liu and Zhao et al., 2022), and they tended to avoid learning with others to avoid being a burden. Over time, most spousal caregivers of older adults with disabilities have developed a stereotype that fosters a fear of technology (Lam and Lu et al., 2020). One similar study outcome revealed that despite the availability of social support, nearly 33 % of senior citizens in the United States were unprepared for video-mediated telehealth consultations (Lam and Lu et al., 2020). De Santis KK et al. noted that many older adults face technological challenges when using telehealth due to age, health-related barriers, or low technological literacy (Ha and Ho et al., 2023). However, family caregivers face these challenges but also have some coping measures to bridge the digital divide, including interactions with peers, other family caregivers, and intergenerational relationships (Cheng and Lyu et al., 2021). In addition, older adults who receive support, coaching and training are more likely to express positive associations with telehealth (Reiners and Sturm et al., 2019). Specific examples of successful training and support include addressing discouragement and inexperience through the provision of initial and ongoing coaching by dedicated coaches, helping to build trust, encouragement and motivation, and involving caregivers in initial training sessions so that they can provide informal ongoing support (Hawley and Genovese et al., 2020).

Preferences of family caregivers of elderly individuals with disabilities for using telehealth in home care were influenced by the popularity of telehealth promotion. Family caregivers often grapple with a lack of sufficient support and resources, and their capacity to deliver the best possible care might be impaired by restricted access to telehealth information, advice, and expert assistance (Girgis and Abernethy et al., 2015). In a cross-sectional survey study conducted in Italy (Gallè and Oliva et al., 2023), it was found that the availability of telehealth services was known by barely half of the 1002 respondents. Notably, the majority of these respondents were also young individuals equipped with advanced mobile devices and possessing robust access to information. What's more, in the population of family caregivers of the elderly with disabilities, the spouses of the elderly with disabilities, as the main family caregivers (Riffin and Van Ness et al., 2019), often possess mobile phones with limited functionalities. This technological constraint impedes their ability to effectively utilize telehealth services, such as video consultations and online health record access (Ekwegh and Cobb, 2023). Furthermore, these caregivers typically exhibited a limited understanding of telehealth because insufficient dissemination and promotion of information have led to family caregivers of incapacitated elderly individuals being hesitant to share health-related information online, primarily due to concerns regarding privacy and security (Taha and Sharit et al., 2014). At the same time, healthcare professionals are less likely to disseminate knowledge about telehealth to family caregivers of elderly people with disabilities (Liu and Li et al., 2023), which further affects their ability to use telehealth services effectively. That meant the benefits and risk avoidance skills of telehealth services should be further publicized in the future, and healthcare providers and health systems should endeavor to develop consistently their infrastructure (Liu and Li et al., 2023), for instance, increasing font sizes, substituting technical terms with pictures and straightforward language, and simplifying operator procedures and interfaces (Liu and Zhao et al., 2022), to make telehealth more accessible to all individuals. Healthcare professionals need to build strong therapeutic relationships with patients and their caregivers to motivate them to adopt telehealth and use it to support their treatment (Yu and McCann et al., 2021).

Cost is one of the factors influencing whether family caregivers of disabled elders use telehealth. The financial implications and reimbursement policies associated with telehealth have frequently been identified as substantial obstacles to its utilization (Zachrison and Boggs et al., 2020). A systematic review also indicated that 36 % of studies identified the cost of telehealth as a major barrier to its accessibility (Chua and Koh et al., 2022), in line with the results of Ramchandran et al. (Ramchandran and Yilmaz et al., 2020), showing that almost all participants regarded their limited health insurance coverage, fixed income, and limited budget as barriers to performing dilated examinations. In the results of this study, many patients expressed a desire to be informed of the examination costs prior to making a decision and indicated a greater willingness to participate if they had known that their insurance would cover the service. However, current reimbursement rates for telehealth remain suboptimal; for instance, a study on outpatient telehealth reimbursement in Michigan showed that the rates of reimbursement differed greatly, ranging from 0 % to 67 % of the total incurred costs (Lin and Kavousi et al., 2020). In China, telehealth fees and Medicare reimbursement rates exhibited considerable inconsistency, with low reimbursement rates identified as a primary impediment to the advancement of telehealth (Ma and Sun et al., 2022). Considering the substantial evidence supporting the reduction of telehealth expenses and prices, this highlighted the necessity for measures that facilitate telehealth visits and reduce the risk of falling into income poverty, even for high-income countries (Callander and Fox et al., 2019).

The findings are consistent with earlier studies using qualitative methods to examine the preferences of different populations for using telehealth, including convenience (Cook and Randhawa et al., 2016), acceptance of telehealth (Mishuris and Stewart et al., 2015), and so on. Unlike previous studies that focused more on patients (Saeed and Singhal et al., 2024), this study analyzed the factors affecting the use of telehealth from the perspective of family caregivers of elderly people with disabilities. This study contributes to improving support for family caregivers and promoting the use of telehealth for older people with disabilities. However, this research had several limitations. Firstly, our sample was selected from a restricted geographical area, which might restrict the generalizability of our results to other areas or nations with distinct healthcare systems and socio-cultural contexts, and therefore a large-scale study in other regions is needed to make the findings more representative. Furthermore, focus group discussion drawbacks include the fact that some individuals may tend to provide more insight or feedback on specific issues, while others may not comment or actively participate, which may lead to uneven contributions and bias in the data collected. At the same time, a few members may overly influence the discussion and the desire to reach consensus may override critical thinking. Third, group dynamics may influence individual perspectives and what is expressed in discussions. As some respondents were unfamiliar with telehealth, their imagination and shared knowledge may have been influenced by the discussion. Fourthly, participants may respond in socially acceptable ways rather than authentic ways. The type of carer may affect their perception of telehealth. Fifthly, most of the participants in this study were spouses of elderly people with disabilities, therefore, the findings may differ from those of other types of caregivers, such as children of elderly people with disabilities.

## 6 Conclusions

The results of this study suggest that family caregivers of elderly people with disabilities consider complex factors when considering whether to use telehealth for home care. These factors included barriers of carergivers' use of telehealth, such as learning capacity, acceptance of telehealth, differences in treatment modalities, health self-management capability, habituation to medical care, job, cost, limitations, operation experience, quality of care in nursing homes, convenience of medical treatment and popularisation; facilitators of carergivers' use of telehealth, such as convenience and professionalism and authority of healthcare professionals. Our research will contribute to the development of telehealth solutions, aiming to improve the care quality for family caregivers of disabled elders and to improve the quality of life among those elderly individuals with disabilities.

This research not only reveals the real dilemmas and potential dynamics of telehealth application in home care for the disabled elderly, but also provides a new empirical basis for research in related fields. Unlike previous studies focusing on the feasibility of telehealth technology, this study pays more attention to the actual needs and decision-making influencing factors of the users - family caregivers, and fills the gap of research on the application of telehealth in home care scenarios. When developing telehealth solutions in the future, emphasis should be placed on addressing the barriers to use faced by family caregivers.

## Funding

This work was supported by the SIRG-CityU Strategic Interdisciplinary Research Grant (No.7020093); Basic and Applied Basic Research Project of Guangzhou Basic Research Program (No. 2023A04J1918); the University Youth Innovative Talent Project of Guangdong Province (No. 2023KQNCX006); the Guangdong Basic and Applied Basic Research Foundation, China (No. 2024A1515012176); and Nurturing funds for nursing young talents of Jinan University (JHA202306025). and Guangdong Medical Research Fund (B2025097).

## Ethical consideration

Ethical approval for the study was granted by the Institutional Review Board of Jinan University (Reference number: JNUKY-2023–0128).

## Statements and declarations

Not applicable.

## CRediT authorship contribution statement

**Lina Guan:** Writing – review & editing, Writing – original draft, Software, Formal analysis, Conceptualization. **Guijuan Zhang:** Writing – review & editing, Writing – original draft, Resources, Funding acquisition, Data curation. **Yuyang Yi:** Writing – review & editing, Writing – original draft, Software, Methodology, Data curation. **Yaqin Liang:** Writing – review & editing, Resources, Investigation. **Xiaochun Zou:** Writing – review & editing, Resources, Investigation. **Bingsheng Guan:** Writing – review & editing, Supervision, Methodology, Funding acquisition, Formal analysis, Data curation, Conceptualization. **Yanya Chen:** Writing – review & editing, Supervision, Software, Methodology, Investigation, Funding acquisition, Formal analysis, Conceptualization. **Wai-kit Ming:** Writing – review & editing, Supervision, Software, Funding acquisition, Conceptualization.

## Declaration of competing interest

No.
